# Phase Stability Control of Optical Fiber Partial Discharge Ultrasonic Sensing System

**DOI:** 10.3390/s22218495

**Published:** 2022-11-04

**Authors:** Chao Xing, Qian Zang, Ruidong He, Jun Zhao, Lili Wang, Lujian Dai, Rongbin Shi, Sihan Wang, Guoming Ma

**Affiliations:** 1 State Grid Hebei Electric Power Co., Ltd., Electric Power Research Institute, Shijiazhuang 310014, China; 2 State Grid Hebei Electric Power Co., Ltd., Shijiazhuang 310014, China; 3 State Key Laboratory of Alternate Electrical Power System with Renewable Energy Sources, North China Electric Power University, Beijing 102206, China

**Keywords:** partial discharge, phase fading, phase feedback control, ultrasonic detection of partial discharge, acousto-optic modulator

## Abstract

Optic fiber interferometers are highly sensitive ultrasonic sensors for partial discharge detection. However, low-frequency vibration and environmental noise will disturb the sensors in the field, and cause a phase fading suppression effect that reduces sensitivity. This paper analyzed the problems existing in the phase feedback control system based on PZT, and an improved scheme incorporating a high-frequency carrier phase demodulation is proposed. Based on an acousto-optic modulator, the proposed phase feedback control system overcomes the phase fading suppression effect. A test is carried out on an ultrasonic calibration platform and a transformer oil discharge platform. The test results show that the stability of the improved phase demodulation system has been significantly improved, and meets the requirements of field applications. Compared with the signal-to-noise ratio at the time of phase fading of the system before the improvement, the signal-to-noise ratio of the improved system is improved by 69 dB.

## 1. Introduction

Partial discharge detection is an effective means to find insulation defects in power equipment, and is of great significance in terms of improving the operational reliability of power equipment [1,2]. At present, the main detection methods of partial discharge in power equipment are the pulse current method, UHF detection, acoustic measurement, light measurement, the decomposition product method, and so on [3,4]. Among them, the pulse current and UHF detection methods have high sensitivity and mature technology, but poor ability when exposed to anti-electromagnetic interference. The optical measurement and decomposition product methods are still in the laboratory research stage, and are thus difficult to apply to the field. The acoustic measurement method has strong anti-electromagnetic interference ability and is suitable for on-site partial discharge detection in complex electromagnetic environments.

The traditional acoustic measurement uses piezoelectric transducers (PZT) to sense the ultrasonic wave generated by partial discharges in power equipment. However, PZT has a relatively weak sensitivity and is difficult to multiplex, and as such, it struggles to meet the needs of partial discharge detection in power equipment [5]. A variety of optical fiber ultrasonic sensors have been developed rapidly and provides a new technical scheme for partial discharge ultrasonic detection. Optical fiber interferometers have the advantages of high sensitivity and simple sensing structure, and have become a research hotspot in recent years [6,7,8,9].

In 1996, scholars at the University of New South Wales first developed an optical fiber ultrasonic sensor with a 150 m long optical fiber, and successfully detected the ultrasonic wave produced by transformer partial discharge based on Michelson interference sensing system [10]. In 2015, Pepper et al. of the University of Applied Sciences in Berlin studied the optical fiber ultrasonic sensor based on Michelson interference, which is used to detect the internal partial discharge of silicone rubber solid insulation under DC voltage. In the partial discharge test, the interferometric optical fiber sensor has the same detection sensitivity as the pulse current method [11]. In 2016, scholars from Shanghai University constructed a Michelson interference optical fiber ultrasonic sensing system, which successfully detected partial discharge signals in GIS outgoing cables [12]. In 2018, Xu et al. of Xi’an Jiaotong University designed an optical fiber ring sensor structure, and constructed a partial discharge detection scheme based on a Sagnac interferometer. The detection capability was explored through the tip discharge model, and the effects of different length sensors on the detection sensitivity were studied. The above research has realized the detection of partial discharge ultrasonic signals and improved the sensitivity to varying degrees [13]. However, most of the studies focus on the detection effect and sensitivity under the ideal conditions of the laboratory, but not on the stability of the sensing system.

Interferometers based on optical fibers are a type of phase modulation ultrasonic sensor. Their response performance is closely related to the initial phase position [14]. Phase shift caused by the change of external temperature and stress will affect the stability of the sensor. The complex operation environment of power equipment and the electromagnetic force caused by switch action and operation current will lead to the shell vibration of power equipment. Under the electromagnetic force, the shell of power equipment vibrates stably at 2 times of the power frequency with the acceleration of 0.01~0.1 g (g = 9.8 m/s^2^), and the frequency spectrum is distributed within 1 kHz. The mechanical vibration of the shell caused by knife switching is larger, and the frequency spectrum distribution is about 100~1 kHz. Although the vibration frequency is much lower than that of ultrasonic signals generated by partial discharge, the modulation effect of these vibrations is much stronger. These low-frequency interference signals will lead to random changes in the initial phase of the interferometric ultrasonic sensing system, which makes it difficult to work stably [15,16].

In order to solve the problem of the phase fading of interference signals mentioned above, the existing frontier scheme is a solution based on phase tracking feedback. This method detects the phase shift caused by the environmental noise, and then drives the phase modulator to produce the opposite phase shift in the interferometer, so as to counteract the random phase shift caused by noise. But this approach is not a one-size-fits-all approach. Due to unknown reasons, the control deviation gradually accumulates when the system works for a long time, resulting in a slow drift of the system operating point.

In this paper, the phase stability of a partial discharge ultrasonic sensing system based on an optical fiber is studied. The causes of the problems of the existing phase stability control methods are analyzed. At the same time, a new phase stability control scheme is proposed. By introducing frequency shift into the previous scheme, the measured carrier is transferred to a higher frequency, thus avoiding low frequency interferences, such as vibration. The practicability of the scheme is verified by a partial discharge test in a transformer oil discharge platform. Finally, an optical fiber sensing system for partial discharge ultrasonic detection with phase stability control is developed, which is of great significance for on-site inspection.

## 2. Disturbing Mechanism of Optical Fiber Ultrasonic Sensing System

### 2.1. Principle of Ultrasonic Sensing Based on Optical Fiber Interferometer

**Figure 1:**
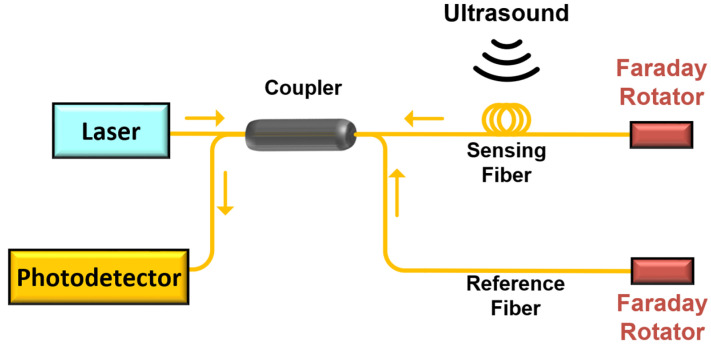
Basic structure based on interference fiber ultrasonic sensing system.

The basic structure of an ultrasonic sensing system based on an optical fiber interferometer is shown in Figure 1. An ultrasonic wave acts on the surface of the sensing fiber in the form of a mechanical wave, which forms acoustic pressure. The acoustic pressure changes the length and refractive index of the sensing fiber, and finally modulates the phase of the transmitted light. Therefore, the external ultrasonic signal can be measured by demodulating the phase shift of the system.

The interference occurs between the sensitive light *E*_1_ and the reference light *E*_2_, and the intensity of the interference light *I* measured by the detector can be expressed as follows [17]:

(1) $$I=I_{DC}+I_{AC}\cos\left(\varphi_{n}+\Delta \varphi \right)$$

where $I_{DC}$ is the DC component of interference light intensity, W; $I_{AC}$ is the amplitude of the AC component of interference light intensity, W; $\varphi_{n}$ is the phase shift of sensing light and reference light caused by external noise interference; and Δφ is the phase shift caused by ultrasonic signal, rad.

It can be seen from Equation (1) that the phase shift of sensing light caused by ultrasound will change the interference light intensity *I*. Therefore, the conventional method is to measure that the amplitude change of *I* can reflect the action of the external ultrasonic wave.

### 2.2. Disturbing Mechanism of Optical Fiber Ultrasonic Sensing System

**Figure 2:**
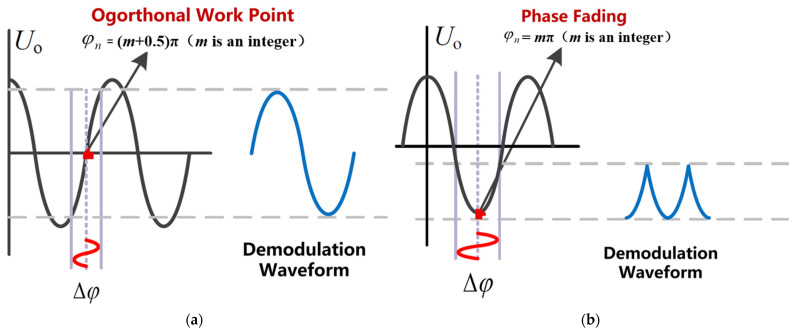
Schematic diagram of the mechanism for affecting the sensing system stability. (**a**) orthogonal work point. (**b**) phase fading work point.

It can be seen from Equation (1) that when the phase is modulated by an ultrasonic wave, the detected signal will be affected by the phase shift $\varphi_{n}$. When $\varphi_{n}$≈ *m*π + 0.5π (*m* is an integer), the AC signal detected in a short time is relatively large because the frequency is much higher, and the system is in the best working state (Figure 2a). However, $\varphi_{n}$ caused by external noise is usually a low-frequency large signal greater than π. When $\varphi_{n}$≈ *m*π (*m* is an integer), the operating point of the system is located at the maximum of the cosine function in a short time (larger than the ultrasonic signal period but much smaller than the noise signal period). This operating point makes the sensitivity of the system decrease sharply. The detected AC signal is very small, which is called “phase fading” (Figure 2b).

In order to ensure the stability and sensitivity of the system, measures must be taken to keep the system functioning at the orthogonal working point.

## 3. Analysis of Problems Existing in Anti-Disturbing Methods of Existing System

### 3.1. Existing Anti-Disturbing Scheme of Optical Fiber Ultrasonic Sensing System

**Figure 3:**
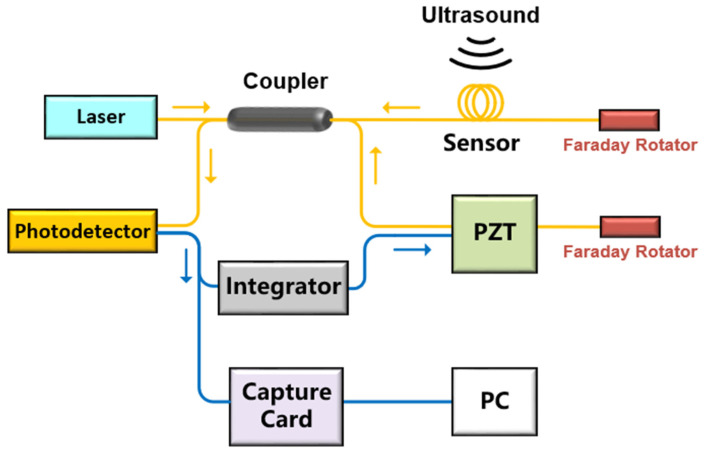
The structure of the interference ultrasonic sensing system with phase feedback control loop.

The structure of the interference ultrasonic sensing system with a phase feedback control loop is shown in Figure 3.

The output voltage of the photodetector is the same as Equation (1). When the change of $\varphi_{n}$ makes the interferometer deviate from the orthogonal working point, the DC component of the output voltage of the photodetector is fed back to the PZT by setting the appropriate integral parameters. Then, the PZT produces the modulation phase shift A(t) and keeps $\varphi_{n}-A(t)=\pi /2+m\pi$ (*m* is an integer), so that the interferometer works at the orthogonality quadrature working point.

### 3.2. Analysis of Problems Existing in Anti-Disturbing Methods of Existing System

However, the above scheme is not perfect. In our study, we found that the system shown in Figure 3 cannot completely avoid phase fading after working for a long time. We find that the hysteresis characteristics of PZT could lead to the slow shift of the working point of the system.

**Figure 4:**
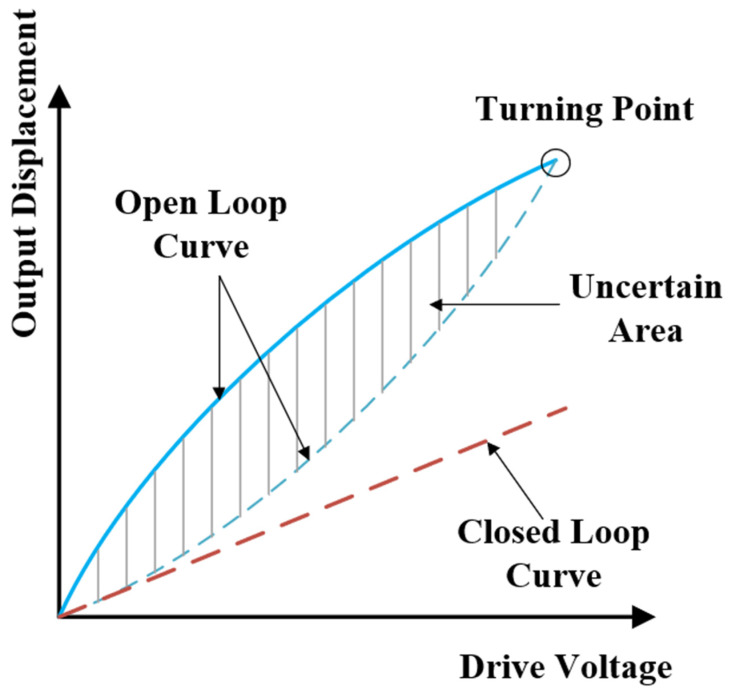
Chart of hysteresis nonlinearity of piezoelectric ceramics.

The main material of PZT is piezoelectric ceramic, and the hysteresis characteristic is the inherent property of piezoelectric ceramics. The hysteresis nonlinear characteristics of electronic ceramic materials are shown in Figure 4. Negative voltage cannot be added to piezoelectric ceramics, so in the maximum input voltage region [0, *U_n_*], the open-loop ascending hysteresis curve (dotted line) and open-loop falling hysteresis curve (solid line) do not coincide. A hysteresis loop (shadow) surrounded by an uncertain region is formed. In the uncertain region, when the input voltage is arbitrary, the rising curve and falling curve corresponding to the input voltage are the upper and lower limits of the piezoelectric ceramic displacement [18].

Usually, there are two kinds of piezoelectric ceramic controllers: open-loop control and closed-loop control. In the open-loop control, the reference displacement is directly inputted into the controller as voltage, which is amplified by the driving module and then pressurized to the piezoelectric ceramic. This control method is simple in structure and convenient to control, but the hysteresis characteristics will greatly affect the accuracy of the system. The closed-loop control frame uses a displacement sensor to realize the closed-loop negative feedback, which has a strong anti-disturbing ability. The gap between the actual output and the expected displacement output corresponding to the reference input is calculated, and then the feedback loop is used to modulate the input. It can achieve high precision control, but greatly reduces the response ability of piezoelectric ceramics.

**Figure 5:**
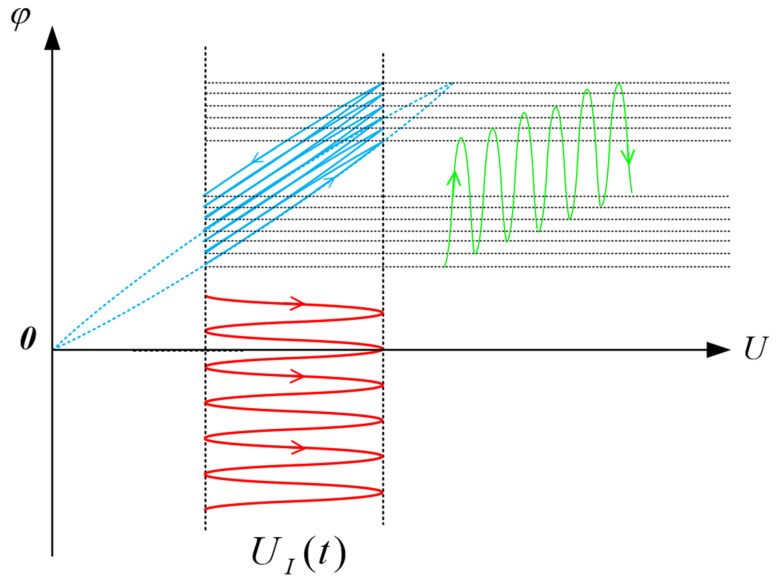
Modulation curve (phase drift) under hysteresis nonlinearity.

Because the interferometric ultrasonic sensing system requires high response ability, the open-loop control mode based on PZT is usually used to adjust the piezoelectric ceramics. However, PZT introduces the contradiction between response ability and control precision into the system. The hysteresis nonlinearity of the piezoelectric ceramics will affect the ultrasonic measurement system. The analysis is as follows:

When a sinusoidal disturbance signal is applied to the piezoelectric ceramic, the modulation curve, as shown in Figure 5, considers the hysteresis nonlinear characteristic of the piezoelectric ceramic. There is a phase shift in the piezoelectric ceramic phase modulator. With the red sine curve pressurizing the piezoelectric ceramics, the voltage-phase modulation curve gradually shifts upward. As a result, the phase modulation is more and more different from that of the first waveform at the same input voltage, and the phase drift occurs in the system. In practical use, the stability of the interferometric ultrasonic sensing system will be affected by both time and external noise. The longer the working time is, the greater and more frequent the external noise is, and thus the worse the phase modulation effect of the phase modulator will be. The working error of the phase modulator makes the system unable to work normally at the orthogonal operating point. The stability of the system is greatly reduced, which brings great challenges to the practical application of system engineering.

## 4. Improved Anti-Disturbing Scheme Based on High Frequency Carrier Phase Demodulation

### 4.1. Improved Anti-Disturbing Scheme Basd on High Frequency Carrier Phase Demodulation

**Figure 6:**
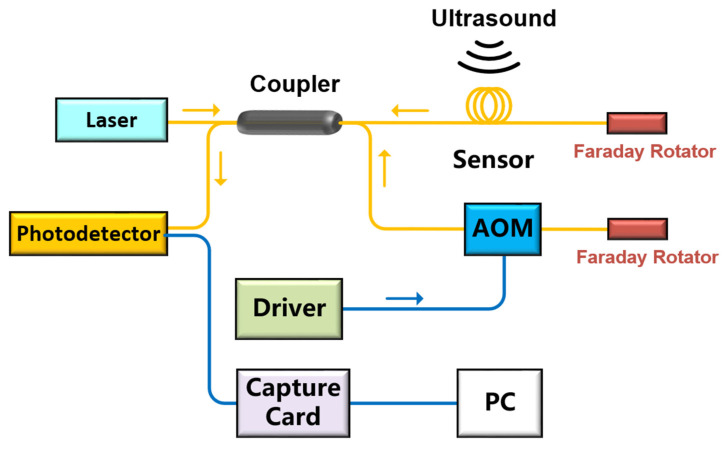
The structure of the interference ultrasonic sensing system based on an AOM high frequency carrier phase demodulator.

An acousto-optic modulator (AOM) is introduced into the reference fiber of the system as a high frequency carrier phase demodulator. As shown in Figure 6, the reference light produces a fixed frequency shift of 200 MHz. At this time, Equation (2) becomes:

(2) $$I^{\prime }=I_{DC}+I_{AC}\cos\left(2\pi f_{1}t+\varphi_{n}+\Delta \varphi \right)$$

where *f*_1_ = 200 MHz is the fixed frequency shift introduced by the AOM.

It can be seen from (2) that the interference light intensity *I’* is a high frequency vibration signal with frequency *f*_1_, and the phase shift Δφ of the sensitive light will affect the phase of the high frequency vibration signal. The shift of the working point of the system will change the amplitude of the signal at the frequency *f*_1_, but has no effect on the result of phase demodulation. Therefor the acoustic pressure amplitude of the external ultrasonic signal can be obtained by the phase shift at the *f*_1_, and there is no more phase fading.

### 4.2. Phase Stable Demodulation Mechanism

In order to demodulate Δφ in Equation (2) stably, we utilize a differential cross multiplication algorithm (DCM) [17]. 

Take Equation (2) as the input U, and set the expressions of the orthogonal reference signals U_1_ and U_2_ as follows:

(3) $$U_{1}=C\cos(2\pi f_{1}t+\varphi_{r})$$

(4) $$U_{2}=C\sin(2\pi f_{1}t+\varphi_{r})$$

**Figure 7:**
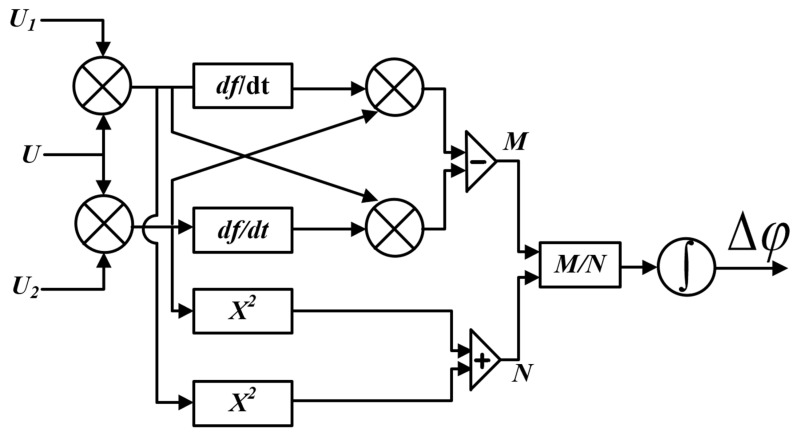
The flow of the DCM algorithm.

Calculated by the DCM algorithm which shown in Figure 7, the time domain signal for the phase Δφ is obtained.

## 5. Test of Phase Fading Suppression Effect

In order to test the phase fading suppression effect of the proposed system, the test is carried out both on an ultrasonic calibration platform and on a transformer oil discharge platform.

The objects of comparison are an ultrasonic sensing system based on an AOM high frequency carrier phase demodulator (Sensing System A) and an ultrasonic sensing system with phase feedback control loop (Sensing System B).

### 5.1. Test on the Ultrasonic Calibration Platform

**Figure 8:**
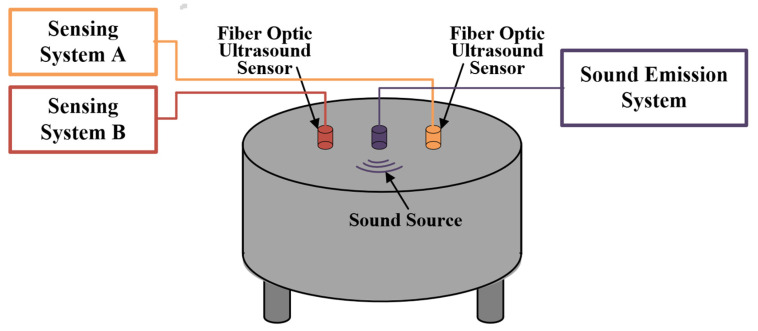
Ultrasonic calibration platform.

The calibration platform of the ultrasonic sensor is established with reference to the national standard GB/T 19801-2005, as shown in Figure 8. The platform uses a cylindrical steel block with an uniform internal structure as the ultrasonic transmission platform, and the acoustic emission PZT is placed in the center of the platform to emit standard sinusoidal signals with equal amplitude and adjustable frequency to simulate partial discharge ultrasonic signals. The optical fiber ultrasonic sensing unit is arranged at the isometric distance (10 cm) on both sides of the acoustic emission PZT. Because the position sound pressure is consistent with the equidistant position of the sound source, the response of the two sensing systems can be compared under the same sound source excitation.

**Figure 9:**
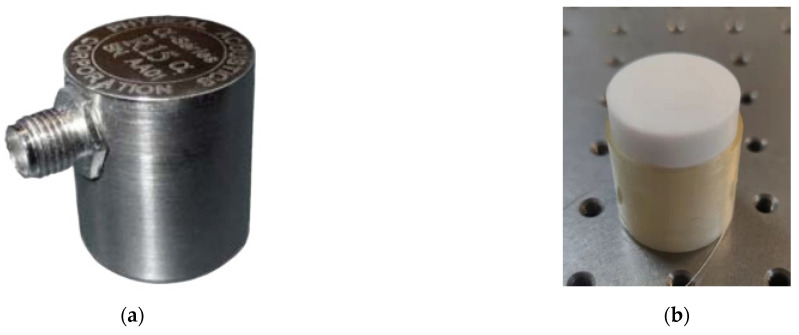
(**a**) Sound source PZT; (**b**) optical fiber ultrasonic sensor.

On the ultrasonic sensor calibration platform shown in Figure 8, the ultrasonic source PZT is the Physical Acoustics R15a (MISTRAS, Princeton Junction, NJ, USA). The ultrasonic sensor is formed by a 50 m optical fiber wound around a polytetrafluoroethylene cylinder. The physical picture of the sound source PZT and the optical fiber ultrasonic sensor are shown in Figure 9.

**Figure 10:**
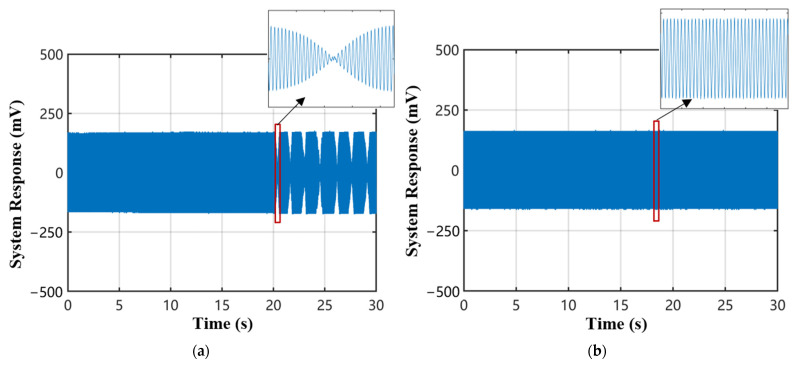
Demodulated signal before and after topology improvement of sensing system. (**a**) Demodulated signal before improvement. (**b**) Demodulated signal after improvement.

The acoustic source PZT continuously emits ultrasonic signals of a fixed amplitude and frequency. The measurement results of the two systems are shown in Figure 10. 

Figure 10a shows the signal of the feedback control using PZT during long-term measurement, and the phase fading phenomenon will appear again with the passage of time. Figure 10b shows that the improved method proposed in this paper completely solves this problem and can obtain a smooth sinusoidal signal. Compared with the signal-to-noise ratio at the time of phase fading of the system before the improvement, the signal-to-noise ratio of the improved system is improved by 69 dB.

### 5.2. Test of Partial Discharge in Transformer Oil

**Figure 11:**
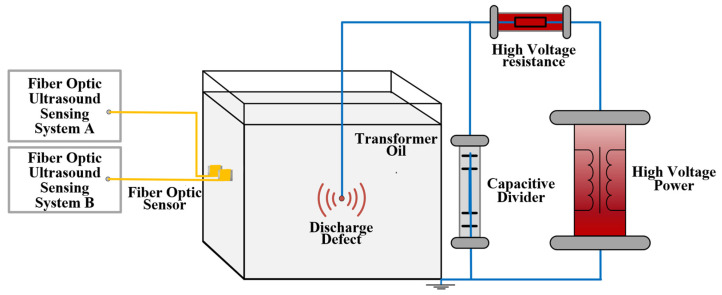
Schematic diagram of partial discharge detection in oil based on optical fiber ultrasonic sensing system.

In order to verify the improved optical fiber ultrasonic sensing system, a test platform for partial discharge detection in transformer oil is built, as shown in Figure 11.

The 25# transformer oil is loaded into the steel transformer oil tank and the suspension discharge model is preset in the oil. Two cylindrical mandrel fiber optic ultrasonic sensors are installed on the outer wall of the fuel tank using the same torque through the magnet adsorption device. The two sensors are connected to the above two different sensing systems respectively.

The operation of the step-up transformer gradually increases the power frequency test voltage, resulting in suspension discharge in the transformer oil tank.

**Figure 12:**
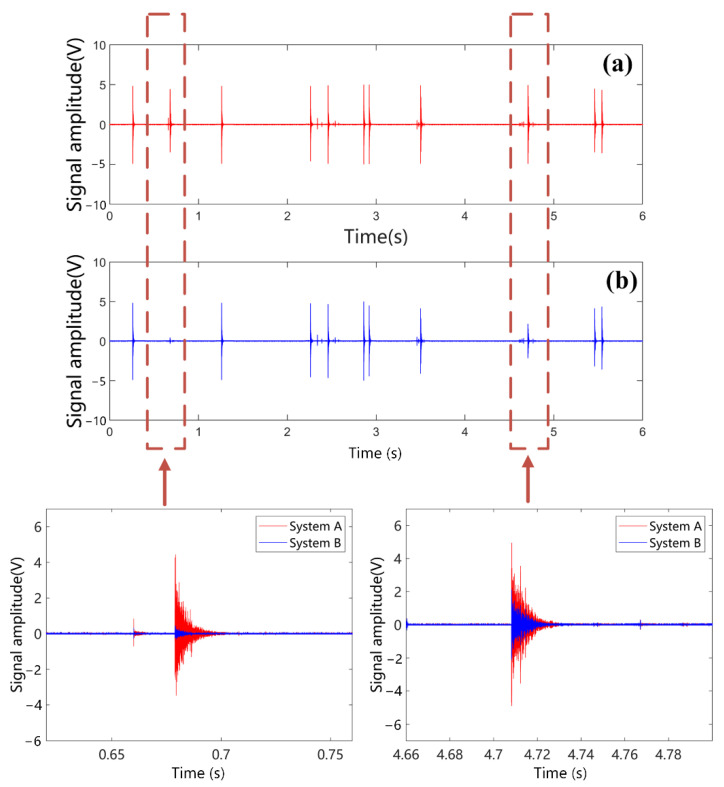
Measurement results of different systems: (**a**) sensing system A (after improvement) (**b**) sensing system B (before improvement).

During the experiment, the step was 2 kV and the voltage was kept at 1 min after each step. The signals detected by the two sensors at each voltage level were recorded until the partial discharge was detected. When the applied voltage gradually increases to a peak of 63.5 kV, the time domain signal is detected, as shown in Figure 12. Several partial discharges were detected by the optical fiber ultrasonic sensing system within 10 s.

It can be seen in Figure 12 that the improved system measured more discharge times, while the system based on PZT phase feedback control lost part of the PD signal due to phase fading. The test results of the ultrasonic calibration platform and the transformer oil discharge platform show that the improved system’s phase demodulation is more stable. For example, at *t* = 0.67 s, the partial discharge signal measured by system A is 15 times of the amplitude of system B

## 6. Conclusions

An optical fiber sensing system for partial discharge ultrasonic detection with phase stability control is developed in this paper. The main conclusions are as follows:

(1) The influence mechanism of environmental noise on the sensitivity of interferometric optical fiber ultrasonic sensing system is revealed and the problems in the existing phase control methods are pointed out. We discovered that the hysteresis characteristics of PZT could lead to the slow shift of the system working point, eventually causing phase fading.
(2) An improved scheme of high frequency carrier phase demodulation based on an AOM is proposed. An acousto-optic modulator is introduced into the reference fiber of the system as a high frequency carrier phase demodulator, and the phase signal is demodulated by a differential cross multiplication algorithm, which effectively suppresses phase fading.
(3) The calibration platform of the ultrasonic sensor is established with reference to the national standard, and calibration experiments were then conducted. Compared with the signal-to-noise ratio at the time of phase fading of the system before the improvement, the signal-to-noise ratio of the improved system is improved by 69 dB.
(4) A test platform for partial discharge detection in transformer oil is built. The measurement results show that the improved system measured more discharge times, while the system based on PZT phase feedback control lost part of the partial discharge signal due to phase fading. The improved system is more suitable for actual engineering measurements.

## Data Availability

Not applicable.
